# Second line use of Fingolimod is as effective as Natalizumab in a German out-patient RRMS-cohort

**DOI:** 10.1007/s00415-013-7082-0

**Published:** 2013-09-06

**Authors:** Stefan Braune, M. Lang, A. Bergmann, NTC Study Group

**Affiliations:** 1 NeuroTrans Concept, Bahnhofstr. 103b, 86633 Neuburg, Germany; 2 Neurozentrum Prien, Bernauer Str. 12, 83209 Prien, Germany

**Keywords:** RRMS, Fingolimod, Natalizumab, Efficacy

## Abstract

Although Fingolimod is registered as a second-line drug in relapsing-remittend multiple sclerosis (RRMS) in Europe there are no clinical studies available comparing Fingolimod (FTY) and Natalizumab (N). This observational cohort-study used health data routinely collected in outpatient neurology practices throughout Germany completing a treatment period of 12 months included 237 patients starting on N and 190 patients on FTY because of failure of the first-line treatment. Mean relapse rate drastically decreased in both treatment groups within three months of therapy in a similar degree and remained on a low level. Both treatment groups saw a similar proportion of patients with unchanged and improved EDSS (80.53 % in FTY, 79.32 % in N). There was no statistically significant difference between the proportion of patients being relapse free (75.79 % in FTY, 71.73 % in N), progression free (87.39 % in FTY, 82.70 % in N) or relapse and progression free (71.05 % in FTY, 62.03 % in N) at 12 months in both strata. Clinical efficacy of FTY and N in RRMS second-line-therapy was similar during the first 12 months of treatment.

## Introduction

Clinical studies of the efficacy of Fingolimod (FTY) investigated FTY as first line medication in relapsing-remitted multiple sclerosis (RRMS) in comparison to placebo [1] or once weekly intramuscular interferon-β-1a [2] showing its superiority regarding relapse rate, progression of disability and end points of MRI. While the United States Food and Drug Administration (FDA) and other countries registered FTY as first-line therapy in RRMS, FTY was registered in Europe by the European Medicines Agency (EMA) only as a second line therapy in RRMS. So far the only registered second line medication in RRMS in Europe had been Natalizumab (N). Clinical trials with N showed its superiority in comparison with placebo [3] and with ongoing interferon therapy alone [4]. This is the first study comparing clinical efficacy of FTY as a second line drug in RRMS with N in a real-life cohort.

## Methods

This is an observational cohort-study using health data routinely collected in outpatient neurology practices throughout Germany who are members of the NeuroTransConcept network. Sex, age, relapses, EDSS and medication are documented digitally in-time during clinical visits at least once within 3-month periods in all patients with MS in the participating practices. All neurologists are trained to document these data in a standardized way in the digital data source and are certified EDSS-raters. This data acquisition protocol is approved by the ethical committee of the Bavarian Medical Board (Bayerische Landesärztekammer, 14.06.2012).

The data of the participating neurology practices are pooled anonymously to form the database of the study. This cohort analysis includes all RRMS patients starting on either FTY or N in the two years between 1 February 2009 and 31 January 2011 and who completed at least 12 months of treatment by 31 January 2012. The decision to change from first-line to second line therapy and the choice of treatment were at the discretion of the treating neurologist and the patient in accordance with the label of the two second-line therapy drugs in Germany.

The primary outcome parameters were progression, as measured by worsening in the EDSS and relapse rate during the first 12 months of treatment with either N or FTY. Differences between treatment groups for demographic parameters, EDSS, relapse rate were tested with a *t* test. Kaplan–Meyer survival curves were calculated analyzing the proportion of patients without progression of EDSS, new relapses as well as for the composite parameter freedom of clinical disease activity, combining lack of relapse and progression. Progression of EDSS was defined as an increase of the EDSS score by one point, if baseline EDSS was smaller than 5.5, or 0.5 points if baseline EDSS was equal or higher than 5.5.

## Population

Two hundred and thirty-seven patients starting on N and 190 patients on FTY were identified and included. Their demographic characteristics are shown in Table 1.

**Table 1:** Demographic data of patient groups

Parameter	Natalizumab	Fingolimod	*p* value
Number of patients fully documented	237	190	
Mean age (years)	37.39 ± 9.6	40.47 ± 8.71	0.0007
Sex—female/male percentage	69.62/30.38	67.89/32.11	0.7028
Mean duration of MS (years)	9.15 ± 6.9	9.89 ± 6.9	0.3577
Mean EDSS at baseline	3.3 ± 1.8	2.3 ± 1.6	<0.0001
Mean annualized relapse rate	0.42 ± 0.84	0.34 ± 0.69	0.3083
Mean relapses 3 months prior to treatment	0.56 ± 0.8	0.25 ± 0.6	<0.0001

Patients with FTY had a significantly lower EDSS at baseline and less relapses during the three months prior to treatment than N, while the annualized relapse rate did not differ significantly between groups.

## Results

**Fig. 1:**
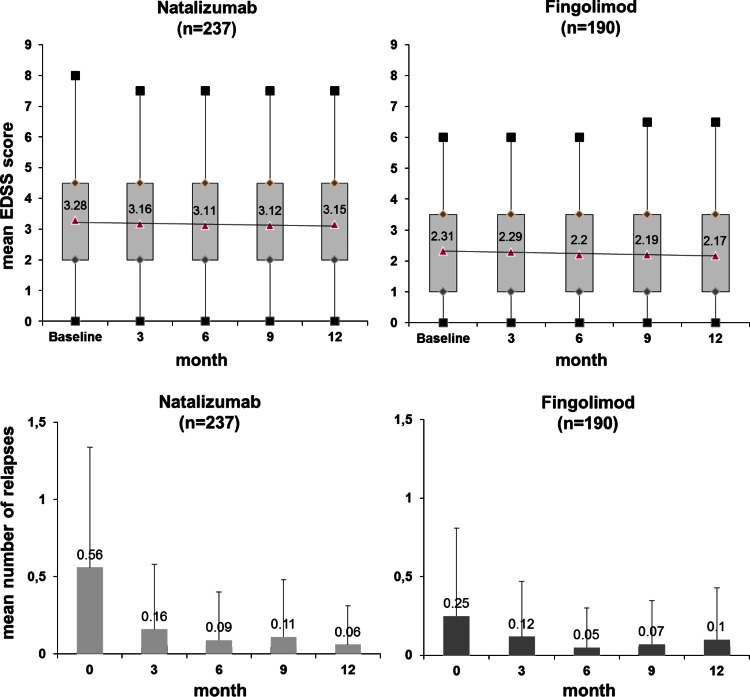
Mean EDSS score and relapse rate in three months intervals of therapy

Mean EDSS scores in both treatment groups showed a slight tendency to improve, without statistically significant change over time within groups or difference of change between groups (Fig. 1).

Mean relapse rate decreased drastically in both treatment groups within three months of therapy in a similar degree and remained on a low level over the documented observation time (Fig. 1).

**Fig. 2:**
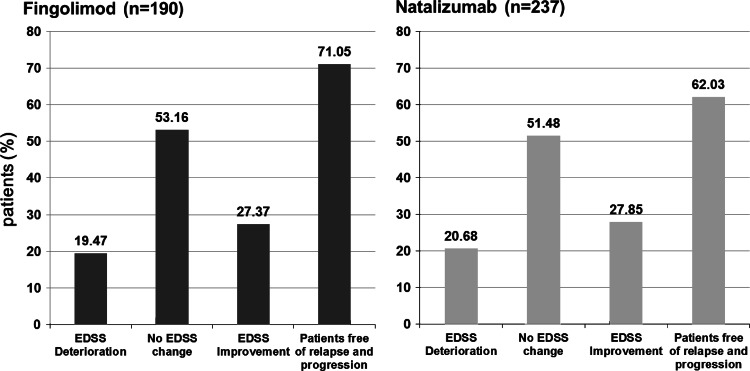
Number and percentages of patients in EDSS strata and free of clinical disease activity

Both treatment groups saw a similar proportion of patients with unchanged and improved EDSS (80.53 % in FTY, 79.32 % in N) and deteriorated EDSS. The higher proportion of patients free of clinical disease activity in FTY was not statistically significant compared to N (Fig. 2).

**Fig. 3:**
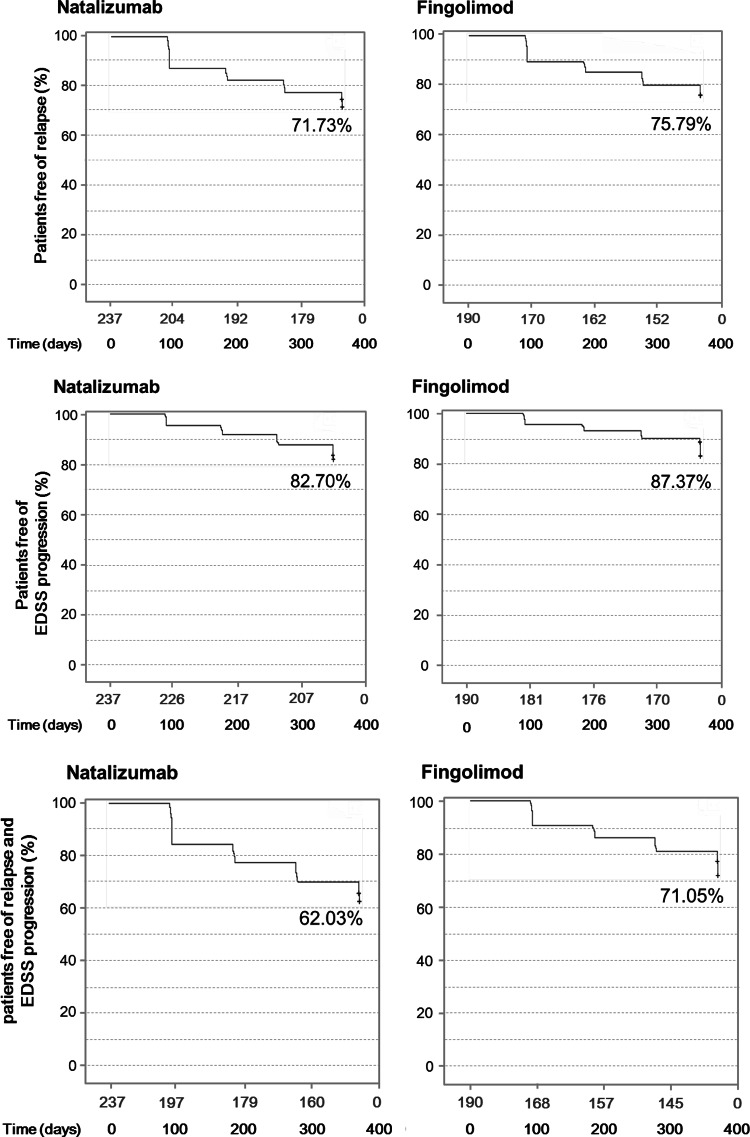
Kaplan–Meyer survival curves of patients free of relapse, free of progression, with freedom of clinical disease activity over one year of therapy with N or FTY

Both treatments achieved a similar proportion of patients being relapse free (*χ* ^2^ *p* value 0.35), progression free (Chi square *p* value 0.18) or relapse and progression free (*χ* ^2^ *p* value 0.05) at 12 months (Fig. 3).

To address potential effects of different baseline demographics of both cohorts, adjusted linear regression analyses have been performed. All clinical and demographic baseline parameters revealed low and non-significant correlation coefficients for the outcome parameter EDSS progression (proportion of patients without progression). Regarding the outcome parameter relapse activity between months 9 and 12, age, EDSS at baseline and number of relapses at baseline revealed weak but significant correlations (see Table 2). These weak correlations account for 3.2 % of the variance at most.

**Table 2:** Spearman correlation coefficients

Parameter	Age	Sex	EDSS baseline	Relapse rate baseline	Therapy FTY vs. N
12 months	*r* value	*r* value	*r* value	*r* value	*r* value
EDSS progression	0.038	0.046	−0.017	0.017	0.065
Relapse probability	0.149*	−0.010	−0.136*	−0.179*	0.046

* *p* < 0.05

To balance observed covariates between subjects from this observational study we employed the propensity score method in addition. In brief, “propensity score stratification” was performed according to the method published by Rosenbaum and Rubin [5]. The same baseline characteristics (age, sex, treatment group, relapses at baseline, EDSS score at baseline), as considered in the adjusted regression model, were also considered in the propensity score stratification method. For each covariate one could see the reduction in imbalance produced by the propensity score. There appeared to be no indication of significant residual imbalance.

The results of the propensity score regression analysis (see Table 3) are fully in line with the adjusted linear regression model already presented in the paper. No significant effect of baseline differences on clinical parameters, like relapses and progression, was found. This is not surprising as this fact has already been stated by Senn et al. [6].

**Table 3:** Propensity score method to analyze influence of baseline characteristics

	Odds ratio	95 % CI	*p* value
Summary of estimated odds ratios for relapses			
PS regression (continuous)	1.39	(0.84, 2.29)	0.20
Summary of estimated odds ratios for EDSS progression			
PS regression (continuous)	0.74	(0.41, 1.34)	0.32
Summary of estimate odds ratios for relapses or EDSS progression			
PS regression (continuous)	0.94	(0.60, 1.47)	0.78

## Discussion

Both primary outcome parameters EDSS and quarterly relapse rate stabilized and improved, respectively, within three months after initiating treatment with FTY or N to a similar extent. Both parameters remained stable over 12 months observation time. The differences in baseline clinical and demographic parameters between treatment groups in this real-life dataset reflect the change in attitude of neurologists and MS patients to optimize therapy at an earlier stage with a lower disability and only single relapses, if a safe and efficient therapeutic option is available. Anyhow, these differences did not influence comparability of the two strata as shown by regression analysis and propensity scores.

These data show that FTY and N positively influence the course of RRMS regarding degree of stabilization of EDSS and reduction of relapses at a similar rate. Both drugs achieve freedom of clinical disease activity in about two-thirds of patients whose disease activity had not been sufficiently controlled under first-line medication. These effects seem to be independent of baseline EDSS and relapse rate. Longer observation periods with higher patient numbers will show whether this trend toward improvement of EDSS over 12 months in both treatment groups is real and which patient strata will benefit most. These data underline the importance of an early change in therapy of RRMS if medication with interferons or glatirameracetate does not achieve sufficient control of disease activity.
